# The Psychometric Properties of a New Measure of Sensory Behaviors in Autistic Children

**DOI:** 10.1007/s10803-016-3018-8

**Published:** 2017-02-17

**Authors:** Louise Neil, Dido Green, Elizabeth Pellicano

**Affiliations:** 1 Centre for Research in Autism and Education (CRAE), UCL Institute of Education, University College London, 55-59 Gordon Square, London, WC1H 0NU UK; 2 Centre for Rehabilitation, Oxford Brookes University, Oxford, UK; 3 School of Psychology, University of Western Australia, Perth, Australia

**Keywords:** Sensory Questionnaire, Sensory behaviors, Sensory sensitivities, Autism

## Abstract

Unusual reactions to sensory input became part of the diagnostic criteria for autism spectrum disorder in the DSM-5. Measures accurately assessing these symptoms are important for clinical decisions. This study examined the reliability and validity of the Sensory Behavior Questionnaire, a parent-report scale designed to assess frequency and impact of sensory behaviors in autistic children. The scale demonstrated excellent internal consistency and concurrent validity, and was a better predictor of autistic symptoms than the Short Sensory Profile within a group of 66 school-age autistic children. The scale also successfully discriminated between autistic and typical children of similar age and ability. The Sensory Behavior Questionnaire has potential as a measure of sensory behaviors in children on the autism spectrum.

## Introduction

Atypical sensory experiences are a defining feature of autism (APA 2013). These experiences are diverse, span multiple sensory domains (Kern et al. 2007) and vary widely between and within individuals. Although many autistic people^1^ enjoy aspects of their sensory experiences, they can also be distressing and lead to difficulties in everyday life (Grandin 2009; Leekam et al. 2007; Zachor and Ben-Itzchak 2014). The accurate measurement and treatment of debilitating sensory sensitivities is therefore a priority.

The most frequently used measure of sensory symptoms in autism, the Sensory Profile (Dunn 1999), was developed and tested primarily with a group of 1037 typical children from North America, alongside smaller groups of children diagnosed with particular conditions such as autism and ADHD, with the aim of identifying sensory processing difficulties within a classroom context. The short version of the scale, the Short Sensory Profile (SSP: McIntosh et al. 1999) has been shown to discriminate autistic and typical children. For example, in one study, 5- to 8-year-old autistic children were rated as having significantly more sensory behaviors than their age-matched typical peers (*p* < .001) on 92% of items as well as total and subscale scores (Tomchek and Dunn 2007). Nevertheless, the SSP features few items relating to hyposensitivity (only two items reflecting under-responsivity) and sensory seeking behaviors. Additionally, some sensory responses that are frequently reported by parents of autistic children, such as lack of response to pain, are not detailed in the SSP. Therefore the SSP may not optimally account for the full range of sensory symptoms in autism. The relatively recent recognition of atypical sensory responses and interests as a core feature of autism in the DSM-5 (APA 2013) underscores the importance of developing suitable tools with which to assess sensory behaviors so that appropriate interventions can be identified. At the same time, it is important to recognise that not all sensory behaviors cause distress or difficulty to individuals with autism and their families. Measures that consider the impact of these behaviors on participation in daily life, rather than simply identify the frequency of such behaviors, may also prove useful for clinicians and educators.

The current study assessed the psychometric properties of a new questionnaire designed to address these issues. The Sensory Behavior Questionnaire (SBQ: Green 2009) was initially designed to assess sensory behaviors in individuals with a moderate-to-severe learning disability or pervasive developmental disorder, with or without a physical disability, as both a clinical and research tool. A focus group of expert clinicians developed the items, with the original intention of creating a sensory inventory featuring an item checklist alongside a behavior observation. In line with recommendations for best practice for development of health measurement scales (Streiner and Norman 2003), face validity and expert opinion were used to select items from this development work and create a questionnaire that assessed *both the frequency and impact* of sensory behaviors in a variety of sensory modalities.

The current study tested the reliability and validity of the SBQ as a parent-report measure of sensory behaviors in cognitively-able autistic children. Specifically, we assessed the internal consistency of the SBQ’s frequency and impact subscale and total scale scores, as well as its concurrent, discriminant and predictive validity. We hypothesised that the SBQ would have (1) excellent internal consistency, (2) good concurrent validity with another parent-report measure of sensory sensitivities, the SSP (McIntosh et al. 1999), (3) good discriminant validity with a parent-report measure of anxiety, the Spence Children’s Anxiety Scale (Spence 1988) and (4) good predictive validity, as assessed by its ability to predict diagnostic status (autistic, typical). Given that the scale was developed with individuals with developmental conditions in mind, and includes items relating to impact as well as frequency, we also hypothesised that the SBQ might offer an advantage over the SSP as a measure of sensory behaviors in autism. To investigate this issue, we examined the extent to which scores on the two scales could predict autistic symptoms.

## Method

### Participants

The parents of 66 autistic children (male = 57; female = 9) and 70 typically developing children (male = 36; female = 34) aged from 6 to 17 years matched on age t(134) = 0.88, *p* = .38, and full-scale IQ, t(99)^2^ = 1.01, *p* = .31^3^ (see Table 1 for scores), as assessed by the Wechsler Abbreviated Scales of Intelligence, Second Edition (WASI-II; Wechsler 2011) took part in this study by completing questionnaires. Participants were recruited through advertisements, the Autism Spectrum Database-UK (http://www.ASD-UK.com), mainstream and special schools and parent support groups in the Greater London area. All parents completed a screening measure for autism, the Social Communication Questionnaire (SCQ: Rutter et al. 2003). All autistic children had previously received an independent clinical diagnosis of an autism spectrum condition according to ICD-10 (World Health Organisation 1992) or DSM-IV (American Psychiatric Association 2000) criteria and further met criteria on either the Autism Diagnostic Observation Schedule (ADOS-G: Lord et al. 1999, 2012; ADOS-2:) using the revised algorithm (Gotham et al. 2007, 2008) or the SCQ (see Table 1).

**Table 1:** Descriptives for autistic and matched typically developing children

	Autistic children (n = 66)	Matched typical children (n = 70)
Age (years)		
M (SD)	10.28 (2.50)	9.90 (2.61)
Range	6.82–16.46	6.00–17.59
Verbal IQ^a^		
Mean (SD)	98.33 (16.01)	104.89 (8.83)
Range	57–130	77–126
Performance IQ^a^		
Mean (SD)	101.48 (15.99)	98.21 (11.44)
Range	75–140	74–119
Full scale IQ^a^		
Mean (SD)	99.74 (15.06)	101.87 (8.31)
Range	70–129	78–114
SCQ^b^		
Mean (SD)	23.58 (8.78) n = 65	4.21 (3.53) n = 63
Range	5–46	0–14
ADOS^c^		
Mean (SD)	10.64 (3.79) n = 55	
Range	4–20	
SBQ^d^ total score		
Mean (SD)	212.94 (47.04)	287.91 (22.60)
Range	99–291	171–300
SBQ^d^ frequency score		
Mean (SD)	98.98 (24.35)	141.71 (13.79)
Range	50–141	81–150
SBQ^d^ impact score		
Mean (SD)	113.95 (24.17)	146.20 (9.24)
Range	46–150	90–150
SSP^e^ total score		
Mean (SD)	115.75 (27.33)	164.06 (21.90)
Range	63–181	90–190
SCAS-P^f^ total score		
Mean (SD)	32.29 (19.24)	16.54 (9.96)
Range	6–76	3–42

^a^Verbal IQ, Performance IQ and Full Scale IQ were measured using the WASI-II (Wechsler 2011)

^b^Social Communication Questionnaire (Rutter et al. 2003). A score of 15 or above indicated elevated levels of autistic symptomology

^c^ADOS = Autism Diagnostic Observation Schedule (Lord et al. 1999, 2012). Scores of 7 or above indicate the presence of an ASD

^d^SBQ = Sensory Behavior Questionnaire (lower scores reflect greater levels of sensory behaviors)

^e^SSP = Short Sensory Profile (lower scores reflect greater levels of sensory behaviors)

^f^SCAS-P = Spence Children’s Anxiety Scale (higher scores reflect greater levels of anxiety)

### Measures

#### The Sensory Behavior Questionnaire (SBQ; Green 2009; Gringras et al. 2014)

The 50-item SBQ was uniquely designed to measure both frequency (e.g., ‘How often have you noticed your child show an unusual response to bright lights in the last month?’) and impact (e.g., ‘How much of a problem is it?’) of 25 sensory behaviors in the following domains: auditory processing, visual processing, movement (vestibular and proprioceptive) processing, tactile processing, oral motor (including gustatory and olfactory) processing and general reactions and organisation. The items and their scoring do not divide hyper- from hypo-responsiveness. Rather, the majority of items ask whether the child shows an *unusual response* to a series of different sensory stimuli/environments. Respondents can provide more information by circling the sort of response their child exhibits (e.g., preoccupation/avoidance) and one or more of the examples of that stimulus given e.g., ‘Does your child show an unusual response to bright lights (preoccupation/avoidance) e.g., spotlights, fair ground/neon strip lights?’ There is a parallel design for the frequency and impact scales, with each being scored on a scale from 1 (all the time/an extreme problem) to 6 (never/not at all). In line with the SSP (see below), *lower* scores indicate greater levels/impact of sensory behaviors. Individual frequency/impact items are summed to create total frequency and impact subscale scores (ranging from 25 for the greatest possible frequency/impact of symptoms to 150 for the least possible frequency/impact of symptoms). An overall total score is created by summing the total frequency and impact scales (scores range from 50 to 300).

#### Short Sensory Profile (SSP; McIntosh et al. 1999)

The 38-item Short Sensory Profile is an adapted version of the original Sensory Profile (Dunn 1999) measuring sensory symptoms in seven domains: tactile sensitivity, taste/smell sensitivity, movement sensitivity, under-responsivity/seeking sensation, auditory filtering, low energy/weakness and visual/auditory sensitivity. Parents rate the frequency of each item on a scale from 1 (always) to 5 (never). Scores are summed to create a total score in which lower scores reflect greater levels of sensory sensitivities. SSP total scores can range from a minimum of 38 (greatest frequency of sensory symptoms) to 190 (no sensory symptoms). McIntosh et al. (1999) demonstrated good psychometric properties for the scale, including adequate internal consistency of the total and subscale scores (Cronbach’s alpha ranged from 0.68 to 0.92), good convergent validity with physiological measures and a discriminant validity of >95% in distinguishing children with and without sensory modulation difficulties.

#### Spence Children’s Anxiety Scale: Parent Report (SCAS-P; Nauta 2004; Spence 1998)

The SCAS-P is a 38-item parent report measure of children’s anxiety, adapted from the child version of the Spence Children’s Anxiety Scale (Spence 1997, 1998). Respondents rate the frequency of each item on a 4-point Likert scale ranging from 0 (never) to 3 (always). Responses to each of the 38 items are summed to create a total score, ranging from 0 to 114. Higher scores reflect greater levels of symptoms. The parent version of the scale has been shown to have good psychometric properties including excellent internal consistency (Cronbach’s alpha of 0.89) (Nauta et al. 2004).

### Procedure

This study was part of a larger investigation into sensory differences in autistic children. Children were seen in one or more face-to-face sessions where they were administered the Wechsler Abbreviated Scales of Intelligence, Second Edition (WASI-II; Wechsler 2011) and the Autism Diagnostic Observation Schedule (ADOS-2; Lord et al. 1999, 2012). Parents were asked to complete the Social Communication Questionnaire, the SBQ, the SSP and the SCAS-P. This study was granted ethical approval by the University’s Research Ethics Committee. Written informed consent was obtained from all parents prior to their and their child’s participation.

### Data Analysis

In cases where a participant had data missing, a total score for that particular scale was prorated on an individual basis, using that individual’s completed items. Total scores were not calculated in cases where a participant missed more than 10% of items on a particular scale. Cronbach’s alpha was used to calculate internal consistency and correlation coefficients were performed to assess concurrent validity (SBQ vs. SSP) and discriminant validity (SBQ vs. SCAS-P). We used logistic regression to investigate how well the SBQ could predict children’s diagnostic status (autistic, typical). Finally, we compared the predictive power of the SBQ and SSP by performing hierarchical linear regression analyses with Social Communication Questionnaire (SCQ) scores as the dependent variable and children’s SSP and SBQ scores as predictors.

## Results

### Psychometric Properties

#### Internal Consistency and Association with Background Variables

The SBQ’s internal consistency was excellent—for all 50 items (Cronbach’s alpha = 0.97) and for the frequency (α = 0.93) and impact (α = 0.94) scales separately. There were no significant associations between total SBQ scores and children’s gender (*r* = 0.02, *p* = .90), age (*r* = 0.18, *p* = .15) or IQ (*r* = 0.05, *p* = .68). Total scores were associated with a retrospective measure of autistic symptomatology, the Social Communication Questionnaire (SCQ) (*r* = −0.39, *p* = .001): increased sensory atypical responses to sensory stimuli were associated with greater levels of autistic symptoms. The link between the SBQ and current autistic symptomatology, as measured by ADOS total algorithm scores, did not reach significance (*r* = −0.24, *p* = .08), and there was no correlation with ADOS severity scores (*r* = −0.19, *p* = .16). Similarly, there was no association between SSP scores and ADOS total (*r* = −0.06, *p* = .68) or severity (*r* = −0.04, *p* = .78) scores.

#### Concurrent Validity

Fifty-nine of the 66 parents of autistic children also completed the Short Sensory Profile (SSP). Total SBQ scores showed a strong positive correlation with scores on the SSP (*r* = .79, *p* < .001), indicating good concurrent validity.

#### Discriminant Validity

We assessed the SBQ’s discriminant validity by examining the association between total SBQ scores and SCAS-P scores, completed by parents of 58 autistic children. Total SCAS-P scores showed a moderate correlation with total SBQ scores (*r* = −0.58, *p* < .001); greater levels of anxiety were associated with more sensory behaviors. A similarly strong association was found between total SCAS-P and total SSP scores (*r* = −0.64, *p* < .001).

#### Predictive Validity

To assess the predictive power of the SBQ, we performed a logistic regression with diagnostic status (autistic, typical) as the dependent variable. As shown in Table 2, the logistic regression model was significant, χ^2^ (1) = 99.42, *p* < .001. SBQ scores explained 69% (Nagelkerke R^2^ = 0.69) of the variance in diagnostic group and correctly classified 87.5% of cases^4^. There were significant differences between autistic and typical children on their total SBQ scores, t(92^2^) = 11.95, *p* < .001, *d* = 2.03, and the frequency, t(102^2^) = 12.67, *p* < .001, *d* = 2.16, and impact subscale scores, t(83^2^) = 10.39, *p* < .001, *d* = 1.76, with large effect sizes for each (see Table 1 for means and standard deviations).

**Table 2:** Logistic regression predicting diagnostic group (autism: n = 66; typical: n = 70) from Sensory Behavior Questionnaire scores

Predictor	B	Std. error	Wald χ^2^	*p*	Odds ratio
Sensory Behavior Questionnaire	−0.06	0.01	33.59	<.001	0.94
Constant	16.89	3.04	30.95	<.001	215,80,280.7

#### Comparison with the SSP

To assess whether the SBQ offered predictive power over and above the SSP, we conducted a hierarchical linear analysis on children’s SCQ scores^5^. Autistic children’s SCQ scores did not correlate significantly with age (*r* = 0.21, *p* = .10), gender (*r* = 0.04, *p* = .75) or full scale IQ (*r* = − 0.22, *p* = .07); these variables were therefore not entered as covariates in the analysis. When autistic children’s scores on the SSP were entered in the first step of the model (see Table 3), they significantly predicted children’s SCQ scores (R^2^ change = 0.08, *F* (1, 56) = 5.12, *p* = .03). When their SBQ scores were entered in the second step, they made a small but significant improvement to the model, explaining an additional 7% of the variance in children’s SCQ scores (R^2^ = 0.15, R^2^ change = 0.07, *F* (1, 55) = 4.53, *p* = .04). Moreover, when both scales were entered together in the analysis, only children’s total SBQ scores significantly predicted children’s SCQ scores (β = −0.42, *p* = .04); children’s SSP scores did not (β = 0.05, *p* = .82).

**Table 3:** Short Sensory Profile scores and Sensory Behavior Questionnaire scores as predictors of autistic children’s autistic symptomatology, as measured by the Social Communication Questionnaire

	R^2^	*∆* R^2^	*∆F*	B	Std. error	β
Model 1	0.08	0.08	5.12			
Constant				34.94	4.89	
SSP^a^				−0.09	0.04	−0.29*
Model 2	0.15	0.07	4.53			
Constant				39.12	5.13	
SSP^a^				0.01	0.06	0.05
SBQ^b^				−0.08	0.04	−0.43*

^a^SSP = Short Sensory Profile Scores (lower scores reflect greater levels of sensory behaviors)

^b^SBQ = Sensory Behavior Questionnaire (lower scores reflect greater levels of sensory behaviors)

*p < .05

One of the defining—and unique—features of the SBQ is that it measures not only the frequency of children’s sensory behaviors, but also their impact on everyday life. The SBQ frequency and impact scales were very strongly associated with each other (*r* = 0.88, *p* < .001) with individual correlations between the 25 item pairs ranging from *r* = 0.70 to *r* = 0.90. The frequency and impact scale scores also showed significant associations with the SCQ and SCAS, but not the ADOS (see Table 4).

**Table 4:** Correlations between the frequency/impact subscale scores of the Sensory Behavior Questionnaire and scores on the Social Communication Questionnaire, Autism Diagnostic Observation Schedule and Spence Children’s Anxiety Scale

	SCQ^b^	ADOS total^c^	ADOS^c^ Severity Score	SCAS-P^d^	SSP^e^
SBQ^a^ Frequency Score	−0.40**	−0.22	−0.18	−0.57**	0.78**
SBQ^a^ Impact Score	−0.36**	−0.24	−0.19	−0.55**	0.74**

*p < .05, **p < .01

^a ^SBQ = Sensory Behavior Questionnaire (lower scores reflect greater levels of sensory behaviors)

^b^SCQ = Social Communication Questionnaire (higher scores reflect greater levels of autistic symptoms)

^c^ADOS = Autism Diagnostic Observation Schedule (higher scores reflect greater levels of autistic symptoms

^d^SCAS-P = Spence Children’s Anxiety Scale (higher scores reflect greater levels of anxiety symptoms)

^e^SSP = Short Sensory Profile (lower scores reflect greater levels of sensory behaviors)

## Discussion

Hyper-and hypo-reactivity to sensory stimuli and sensory seeking behaviors are now considered within DSM-5’s restricted, repetitive and stereotypical behaviors domain for autism spectrum disorder. Measuring the nature and impact of sensory behaviors effectively is important for detecting such behaviours and for identifying appropriate therapy programmes. Here, we tested a newly developed parent-report scale, the Sensory Behavior Questionnaire (SBQ) (Green 2009; Gringras et al. 2013), within a group of cognitively-able autistic children. The SBQ showed excellent internal consistency, and good concurrent validity with the Short Sensory Profile (SSP). It also discriminated well between autistic children and typical children of similar age and intellectual ability. These findings demonstrate that the scale has potential as a psychometrically valid tool to assess sensory behaviors in children on the autism spectrum.

Children’s SBQ scores also contributed significant, additional information (7% of unique variance—over and above the SSP), in regard to children’s autistic symptomotology. These results imply that the newly developed scale is at least as good at predicting autistic symptoms on a screening measure for the condition, the Social Communication Questionnaire, as the existing scale (the SSP), and might even have an advantage in capturing autistic children’s sensory experiences. It is noteworthy, however, that the SBQ scores only explained a small amount of the variance in autistic children’s SCQ scores, and did not correlate significantly with their ADOS scores. This is perhaps unsurprising given that the SBQ is a parent-report measure of only one feature of autism, unusual sensory behaviors, and that these and related non-social behaviors may not become apparent during the brief (~40-min) observational ADOS assessment.

The SBQ showed a moderate association with the measure of children’s anxiety, the SCAS-P. Although the magnitude of this association was both similar to the magnitude of the SCAS-P and SSP correlation and consistent with that of previous studies investigating the relationship between sensory behaviors and anxiety (Green et al. 2012; Wigham et al. 2015), it nevertheless raises the issue of whether the SBQ—or any other measure of sensory behaviours—can discriminate fully sensory behaviors from anxiety. Previous studies have shown that anxiety and sensory sensitivities regularly co-occur and may be causally linked (although the precise nature of this link is unclear; see Neil et al. 2016) rendering the use of anxiety as a measure of discriminant validity a potential limitation of this study. It is of course possible that a lack of specificity in questionnaire items used to measure these constructs and/or the use of caregiver report to measure both constructs, might have superficially inflated the association between anxiety and sensory sensitivities here and in previous studies (Green et al. 2012; Wigham et al. 2015).

The Sensory Behavior Questionnaire measures the impact, as well as the frequency, of sensory behaviors. Both scales showed a similarly-sized association with autistic symptomotology and levels of anxiety. In fact, the two scales were exceptionally closely associated with each other, with even the most disparate item pair showing a strong correlation (*r* = 0.70). Nevertheless, this aspect of the questionnaire may prove useful on a case-by-case basis, by providing further justification for the need for intervention and helping guide clinical decisions in regards to where best to direct treatment. Furthermore, the frequency and impact components of the questionnaire may afford occupational therapists providing support for sensory features with useful outcome measures. Future studies should investigate whether the impact scale better predicts children’s day-to-day functioning and outcomes over time using measures of adaptive behavior or quality of life.

In conclusion, we have demonstrated that the freely available SBQ is a psychometrically valid assessment of atypical sensory behaviors in cognitively-able autistic children. Important next steps include assessing the scale’s convergent validity with assessments of sensory reactivity provided through a different type of measure (e.g., observation; see Tavassoli et al. 2016) or by a different rater (e.g., self-report); assessing its test–retest reliability, and examining its ability to discriminate between autistic children and those with other conditions such as ADHD. It may also prove fruitful to examine the factors and clusters within the SBQ within a larger sample, as has been done with the SSP (Lane et al. 2011, 2014) to develop further our understanding of the patterns and subtypes of sensory behaviors within the autism spectrum.

## Footnotes

1 The term ‘autistic person’ is the preferred language of many people on the spectrum (Kenny et al. 2016). In this article, we use this term as well as person-first language to respect the wishes of all individuals on the spectrum.

2 Levene’s test of equal variances was significant, thus we report the adjusted independent t test results where equal variances were not assumed.

3 In the larger typically developing group from which this matched sample was drawn (n = 104; male = 54), there was no significant difference in Sensory Behaviour Questionnaire total scores by gender (t(102) = 0.61, *p* = .54) so the autistic and typical groups were not matched on this variable.

4 We also assessed the predictive power of the SSP in this sample using a similar regression model. SSP scores explained 61% (Nagelkerke R^2^ = 0.61) of the variance in diagnostic group (compared to the SBQ’s 69%) and correctly classified 87.2% of cases, similar to the SBQ’s correct classification of 87.5% of cases, χ^2^ (1) = 66.68, p < .001.

5 We use children’s SCQ scores as the dependent variable in this analysis because these scores (1) were significantly associated with SBQ and SSP scores (ADOS scores were not) and (2) showed greater variation than ADOS scores, making them more suitable for correlational/regression analysis.
